# Optimization of green and environmentally-benign synthesis of isoamyl acetate in the presence of ball-milled seashells by response surface methodology

**DOI:** 10.1038/s41598-023-29568-y

**Published:** 2023-02-16

**Authors:** Amir Hossein Fattahi, Mohammad G. Dekamin, James H. Clark

**Affiliations:** 1 Pharmaceutical and Heterocyclic Compounds Research Laboratory, Department of Chemistry, Iran University of Science and Technology, Tehran, 16846-13114 Iran; 2 Green Chemistry Centre of Excellence, Department of Chemistry, University of York, York, YO10 5DD UK

**Keywords:** Environmental economics, Molecular ecology, Climate sciences, Environmental sciences, Ocean sciences, Solid Earth sciences, Chemistry, Biosynthesis, Catalysis, Chemical biology, Chemical engineering, Energy, Environmental chemistry, Green chemistry, Inorganic chemistry, Materials chemistry, Organic chemistry, Process chemistry, Surface chemistry, Chemical synthesis

## Abstract

Ball-milled seashells, as a nano-biocomposite catalyst and natural source of CaCO_3_ in its aragonite microcrystalline form with fixed CO_2_, was optimized for the synthesis of isoamyl acetate (3-methylbutyl ethanoate) by response surface methodology with a five-level three-factor rotatable circumscribed central composite design. The seashells nano-biocomposite has proved to be an excellent heterogeneous multifunctional catalyst for the green and environmentally-benign synthesis of isoamyl acetate from acetic acid and isoamyl alcohol under solvent-free conditions. A high yield of 91% was obtained under the following optimal conditions: molar ratio of alcohol: acetic acid (1:3.7), catalyst loading (15.7 mg), the reaction temperature (98 °C), and the reaction time (219 min). The outstanding advantages of this protocol are the use of an inexpensive, naturally occurring and easily prepared nano-biocomposite material having appropriate thermal stability and without any modifications using hazardous reagents, lower catalyst loading and reaction temperature, no use of corrosive Bronsted acids as well as toxic azeotropic solvents or water adsorbents, and simplicity of the procedure.

## Introduction

With regarding to environmental concerns and its direct influence on humans and living organisms, the designing, development and application of environmentally friendly and atom efficient chemical from procedures, safely and appropriate catalysts, reagents and solvents has received considerable attention from both academia and industry in line with the principles of green and sustainable chemistry^1–5^. The ball milling technique and using of heterogeneous catalytic systems containing nano-biocomposites and biopolymers are some of these interesting and useful procedures or concepts. The ball milling is an interesting and green mechanical technique in nano-biocomposite preparation. It is an exciting election for the manufacturing of new nanostructured materials from eco-friendly sources compared to conventional methods for preparation of nano-biocomposites. This method has the advantages including the sharp decline of environmental disposal, the synchronous creation and homogeneous dispersion of nanoparticles, coating of inorganic nanoparticles and the possibility of parallel processes (surface grafting, embedding, and polymerization), which are particularly suitable in the case of biodegradable polymers. Furthermore, the possibility of in situ creating of the nanoparticles and promoting chemical reactions between both the organic molecules and activated nanoparticles as well as the use of solvent-free conditions represent another important advantages of this technique^6–10^.

The esterification of carboxylic acids with alcohols is one of the most important, straightforward and challenging reactions from both academic and industrial points of view^11,12^. Esters are mainly produced from the reactions between the corresponding acids and alcohols or alkyl halides that are traditionally using acid or base catalysis conditions, respectively^13^. Indeed, esterification under acidic conditions is typically a reversible and slow reaction, which requires a higher amount of alcohol to be achieved. Hence, in the case of low alcohol concentration, the conversion requires a long time reaction^14,15^. Interestingly, short-chain esters are important organic compounds that are widely used in different fields of chemical industry such as lubricants, plasticizers, pharmaceuticals, cosmetics, beverages, perfumes, solvents, and food preservatives^16–18^. They are commonly produced from short-chain acids and alcohols with the chain lengths of less than 10 carbon atoms^19^. One of these important esters is isoamyl acetate (3-methylbutyl ethanoate), which is widely used in medicinal, cosmetic, perfume, nuts ices, beverage, candies, bakery products, and other food industries. Other applications of this ester are in honey bee farms, as an alarm pheromone, as well as extraction of penicillin^16,19–21^. Furthermore, isoamyl acetate has high antifungal, antibacterial and antimicrobial activity and is effective in inhibiting and deactivating growth of various micro-organisms and yeasts such as *Escherichia coli*^22^. These applications are very important due to global population growth and its food chain. While a large number of commercial esters can be extracted from natural sources or produced by fermentation, products obtained through these methods have low volumes and high prices. Therefore, more convenient and less costly alternative processes including esterification of carboxylic acids are in high demand^23,24^. Esterification of carboxylic acids with alcohols usually involves homogeneous acid catalysts such as H_2_SO_4_, HCl, HF, H_3_PO_4_ and *p*-toluenesulfonic acid via a chemical synthesis path^25^. Although these catalysts are often inexpensive, they have disadvantages such as toxicity, corrosion and difficulty in their separation^26^. In this regard, heterogeneous catalytic systems have emerged as a suitable alternative for the homogeneous ones. They offer a lot of advantages including higher purity of the products, easy separation, recovery of the catalysts and the potential for reactions under solvent-free conditions^27–29^. A literature review shows that various heterogeneous catalytic systems have been presented for the production of isoamyl acetate from acetic acid and isoamyl alcohol. For example, cation exchange resins such as purolite CT-175, Amberlyst-15 or Amberlite IR-120, tungstophosphoric or molybdophosphoric acid supported on zirconia, poly(vinyl alcohol) containing sulfonic acid, Candida antarctica immobilized lipase, Candida antarctica lipase B onto resin Purolite@MN102, Bacillus aerius lipase immobilized on silica gel matrix^30–35^, hybrid membrane process^36^, polyoxometalate-based sulfonated ionic liquid^37^ and β-MnO_2_ nanorods^38^ can be stated. Also, acidic ionic liquids such as 1-sulfobutyl-3-methylimidazolium hydrogen sulfate ([HSO_3_bmim][HSO_4_]), trihexyl(tetradecyl)phosphonium cation and mixed chloride and bis(trifluoromethylsulfonyl)imide anion have been reported^39^. In most of these methods, only kinetic factors for the optimal synthesis of isoamyl acetate have been investigated. Moreover, some of these protocols have difficulties such as high catalyst loading and the use of organic solvents^32,34,35^. On the other hand, a few of these procedures have used experimental design in order to optimize production of isoamyl acetate^30,31^. Along this line, different bioploymeric macromolecules have received a lot of interest, as support, in heterogeneous catalytic systems or in composite materials. In particular, bioploymeric macromolecules such as chitin (poly[β-(1 → 4)-*N*-acetyl-D-glucosamine]; a member of the polysaccharides family), which is ranked as the second most abundant resource after cellulose with an annual production estimated to be of several thousand tones, or its deacylated product (chitosan) are very popular for this purpose^40–46^. Other biopolymers including starch, cellulose, alginates, collagen, fibroin, and wool may demonstrate similar role in the corresponding nano-bicomposite catalytic systems^40,46–57^.

Response surface methodology (RSM) is an efficient statistical method in order to optimize multiple variables to predict optimal conditions with minimum number of experiments. The use of this method, compared to the conventional techniques of one variable at a time, leads to a decrease of time and expense as well as a reduction in the consumption of reagents and materials^16,58–64^.

**Figure 1:**
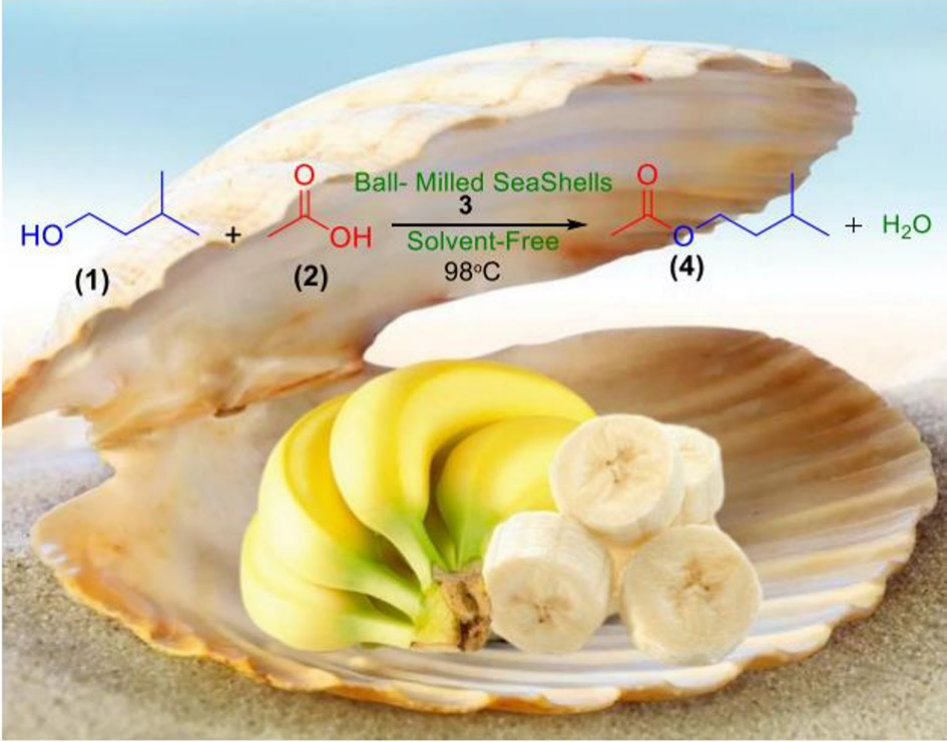
Synthesis of isoamyl acetate (**4**) catalyzed by the ball-milled seashells nanocomposite (**3**) under solvent-free conditions.

In continuation of our interest to explore biopolymeric or nano-ordered catalysts for organic transformation^42–46,54–57^, herein we wish to report ball-milled seashells (**3**), as a nano-biocomposite catalyst and natural source of CaCO_3_ in aragonite microcrystalline form reinforced by chitin fibers and protein chains (3%)^65–67^, for the synthesis of isoamyl acetate (**4**) from isoamyl alcohol (**1**) and acetic acid (**2**) under solvent-free conditions. Moreover, response surface methodology (RSM) with a five-level three-factor rotatable circumscribed central composite design (RCCCD) was performed to estimate the effective parameters including reaction time, reaction temperature and molar ratio of alcohol: acid, and catalyst loading (Fig. 1). To the best of our knowledge, this is the first report on the catalytic activity of the pure ball-milled seashells for organic transformations.

## Results and discussion

### Catalyst characterization

Natural CaCO_3_ has microcrystalline forms such as calcite, aragonite, dolomite or vaterite. Among them, aragonite structure has biocompatibility properties. This microcrystalline form can be found naturally in the crust of all clams and often in bivalves (pH = 10.32). Compared to natural CaCO_3_ particles in the microcrystalline form of calcite (pH = 9.91), seashells are layered. Layers are composed of two parts, an inorganic section inclusive CaCO_3_ and an organic section containing chitin, protein or polysaccharides chains, which make them a natural nano-sized biocomposite. This biocomposite can show its unique catalytic activity for organic transformations when its highly ordered and compact layers are crushed by milling to afford a material offering more surface area. In this study, seashells were collected from the southern coast of Caspian Sea, Babolsar, Iran. The shells were thoroughly washed with distilled water, refluxed in EtOH for 30 min and then oven-dried at 50 °C for 1 h. The obtained dried-seashells were ball-milled in a stainless steel ball mill vessels at 25 Hz and ambient temperature.

**Figure 2:**
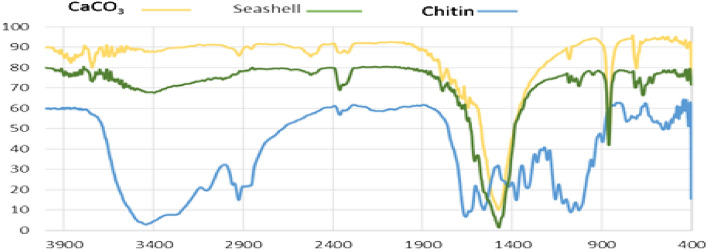
Comparison of FTIR spectra of ball-milled seashells (**3**), commercial calcite CaCO_3_ and chitin.

**Figure 3:**
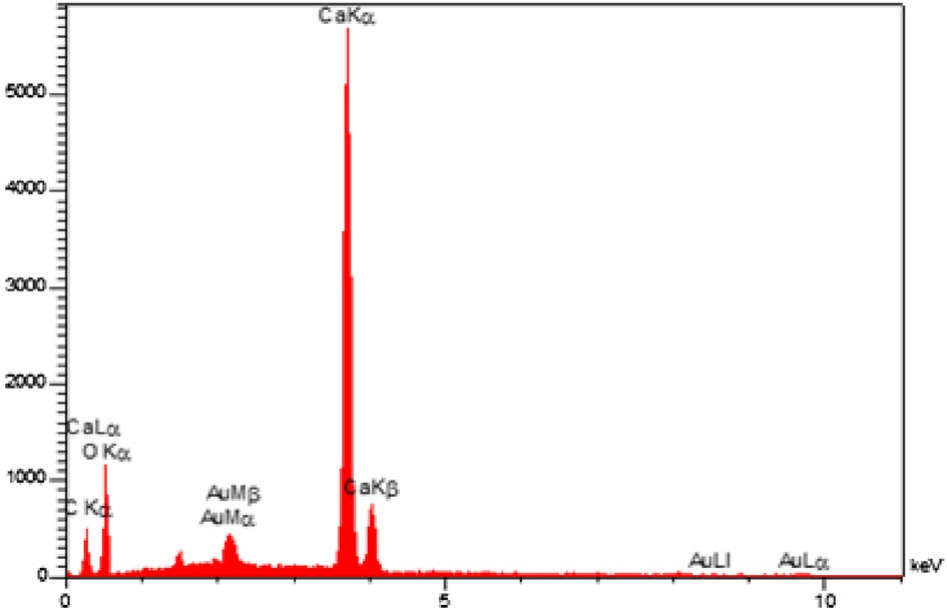
Energy-dispersive X-ray (EDX) analysis of the ball-milled seashells nano-biocomposite (**3**).

**Figure 4:**
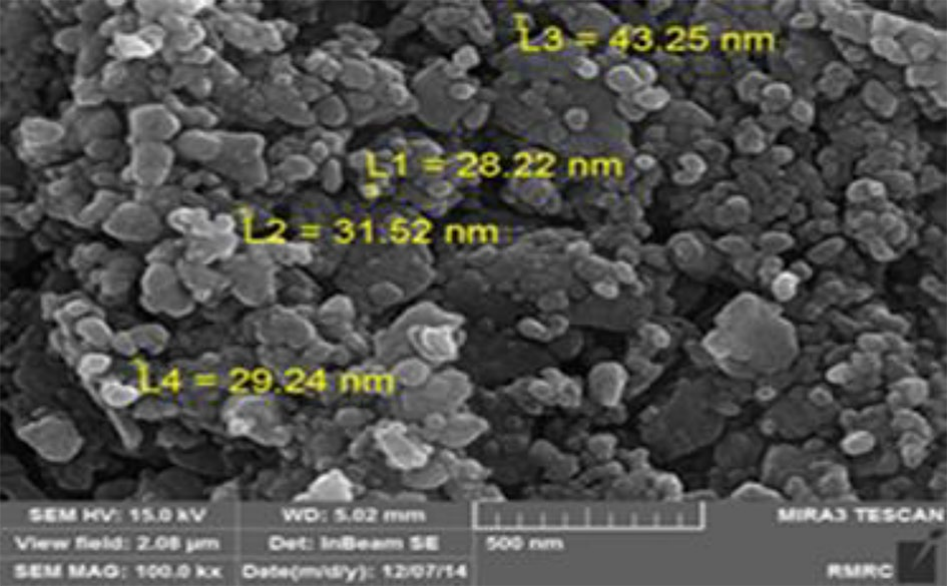
SEM image of the ball-milled seashells nano-biocomposite (**3**).

The FTIR spectra of the ball-milled seashells (**3**), commercial CaCO_3_ and chitin are shown in Fig. 2. The absorption bands observed at 1452, 1081, 840 and 710 cm^−1^ are related to the aragonite CaCO_3_ nanocrystals^65–67^. By comparing of the FTIR spectra of the catalyst and commercial calcite CaCO_3_, it can be found that the biocomposite catalyst **3** is mainly comprised of CaCO_3_. Furthermore, results of energy-dispersive X-ray (EDX) analysis showed that the nano-biocomposite seashells (**3**) includes elements including calcium, carbon and oxygen (Fig. 3). Moreover, the scanning electron microscopy (SEM) image of the catalyst **3** showed an almost uniform spherical particles distribution by the average of particles size about 28–43 nm (Fig. 4).

**Figure 5:**
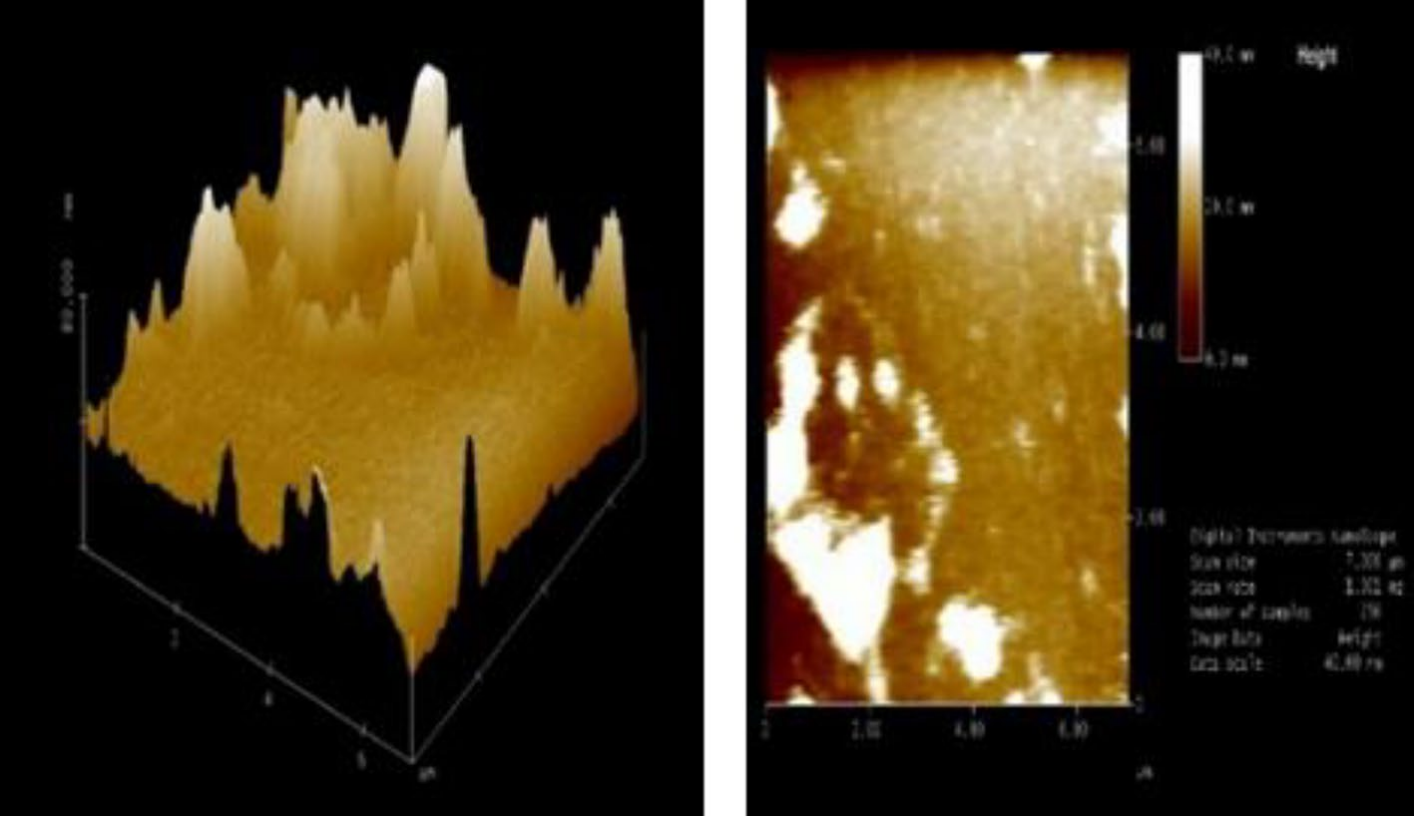
Atomic force microscopy (AFM) of the ball-milled seashells nano-biocomposite (**3**).

**Figure 6:**
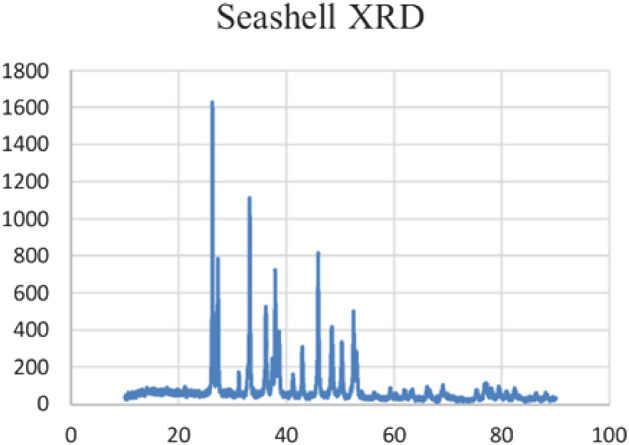
X-Ray diffraction (XRD) pattern of the ball-milled seashells nano-biocomposite (**3**).

Also, the atomic force microscopy analysis of the ball-milled seashell nano-biocomposite shows the topology of the catalyst** 3** surface. AFM images confirms that the catalyst particles are at the nanoscale (Fig. 5). On the other hand, the X-ray diffraction (XRD) pattern of the ball-milled seashells nano-biocomposite (**3**) demonstrated that the crystalline quality of aragonite CaCO_3_ in the obtained powder is maintained throughout the ball milling process (Fig. 6)^65–67^.

**Figure7:**
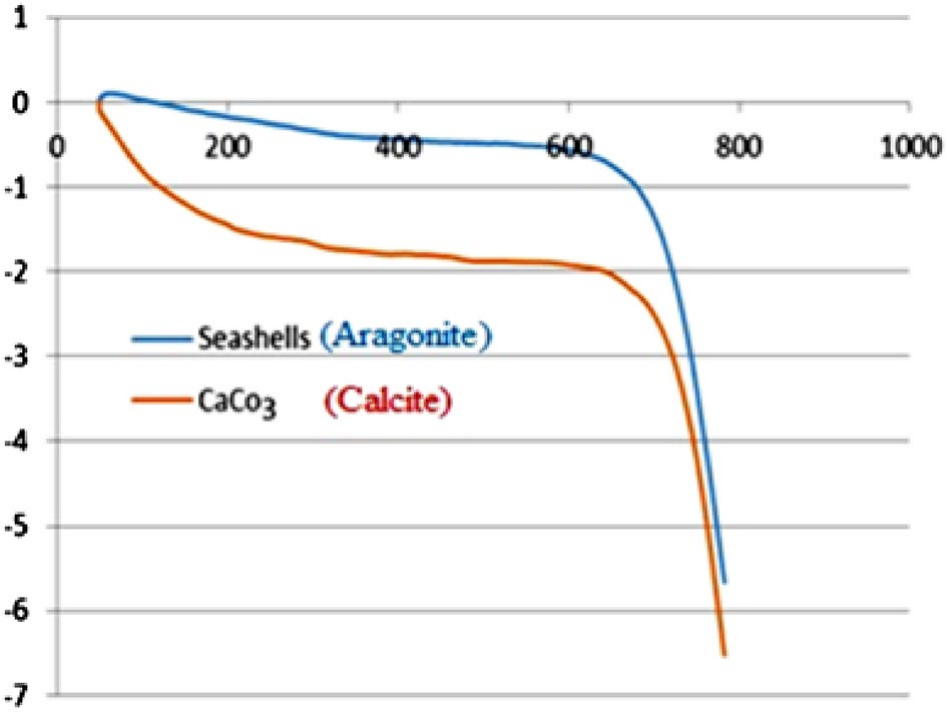
Thermogravimetric (TG) of the ball-milled seashells nano-biocomposite (**3**).

In addition, the thermogravimetric analysis (TGA) of the catalyst **3** demonstrated that significant reduction of the weight of the seashells was not observed until around 600 °C. This indicates that seashells have high thermal stability and more than commercial CaCO_3_ in the calcite microcrystalline from (Fig. 7).

### Optimization of the esterification of isoamyl alcohol (**1**) with acetic acid (**2**) by RSM

Our aim in this research was the improvement of the RSM model for finding the best effective relationship between four variables including reaction time, reaction temperature, molar ratio of acid: alcohol, and catalyst loading. Therefore, analysis of variance (ANOVA) was assessed with regard to significance of the effect of operational parameters and their interactions on the yield of esterification of isoamyl alcohol (**1**) with acetic acid (**2**). The raw isoamyl acetate (**4**) yield data did not well fit to various models (linear, quadratic, and cubic). Accordingly, a convenient data transformation was required^60,68,69^. With this transformation, the experimental data well fit to the quadratic model finally. The model equation, in terms of the variables, is shown in Eq. 1. Generally, it is favorable to fit the lowest order polynomials that sufficiently describe the system. Therefore, a quadratic polynomial model was fitted to obtain the yield of isoamyl acetate (**4**). The quadratic model has been chosen based on stepwise procedure, and the model terms have been selected according to their *p* values (> 0.05). The obtained results of ANOVA analysis are shown in Table 1.

**Table 1:** ANOVA table of the quadratic response surface model.

Source	SS^a^	Df^b^	MS^c^	F-value	Prob > F
Model	9258.76	10	925.88	30.66	< 0.0001 (significant)
A-Temp	5342.73	1	5342.73	176.91	< 0.0001
B-Time	1993.69	1	1993.69	66.02	< 0.0001
C-Cat	99.53	1	99.53	3.30	0.0853
D-Molar ratio of acid: alcohol	341.95	1	341.95	11.32	0.0033
AB	191.20	1	191.20	6.33	0.0210
AC	264.88	1	264.88	8.17	0.0100
AD	278.31	1	278.31	9.22	0.0068
A^2^	308.62	1	308.62	10.22	0.0047
B^2^	119.34	1	119.34	3.95	0.0614
D^2^	575.79	1	575.79	19.07	0.0003
Residual	673.34	19	30.20	–	–
Lack of fit (Not significant)	566.39	14	33.35	1.56	0.3278
Pure Error	106.95	5	21.39	–	–
Cor Total	983,257	29	–	–	–

^a^Stand for sum of square.

^b^Degree of freedom.

^c^Mean of source.

The ANOVA analysis, as shown in Table 1, confirmed the adequacy of quadratic model to demonstrate the actual relationship between the response and the significant variables, since the probability value was lower than 0.0001. Moreover, it can be seen from Table 1, the F value of 30.66 indicates that the above mentioned model is significant. On the other hand, the calculated F value of lack of fit of 1.56 indicated that it was not significant relative to the pure experimental error and confirms the reliability of the model. The *p* values from Table 1 were used to check the significance of each of the factors and interaction between them. The value of prob F less than 0.05 for a variable implies that its effect is significant at the 95% of confidence interval^16,70,71^. Values greater than 0.1 display that the variable is not significant. Hence, the effects of terms A, B, C, D, AB, AC, AD, A^2^, B^2^ and D^2^ are significant to explain the model.

The model created was in the coded format and is shown in Eq. 1. Thus, the final second-order polynomial equation is:

1 $${\text{Yield }} = + {48}.{24} + {13}.{69}*{\text{A }} + { 8}.{36}*{\text{B }} + { 1}.{87}*{\text{C }}{-}{ 3}.{46}*{\text{D }} + {3}.{46}*{\text{A}}*{\text{B }}{-}{ 3}.{93}*{\text{A}}*{\text{C }} - {4}.{17}*{\text{A}}*{\text{D }} + { 2}.{22}*{\text{A}^{2 }} + {1}.{38}*{\text{B}^{2 }} + {3}.0{3}*{\text{D}^{2}}$$

**Figure 8:**
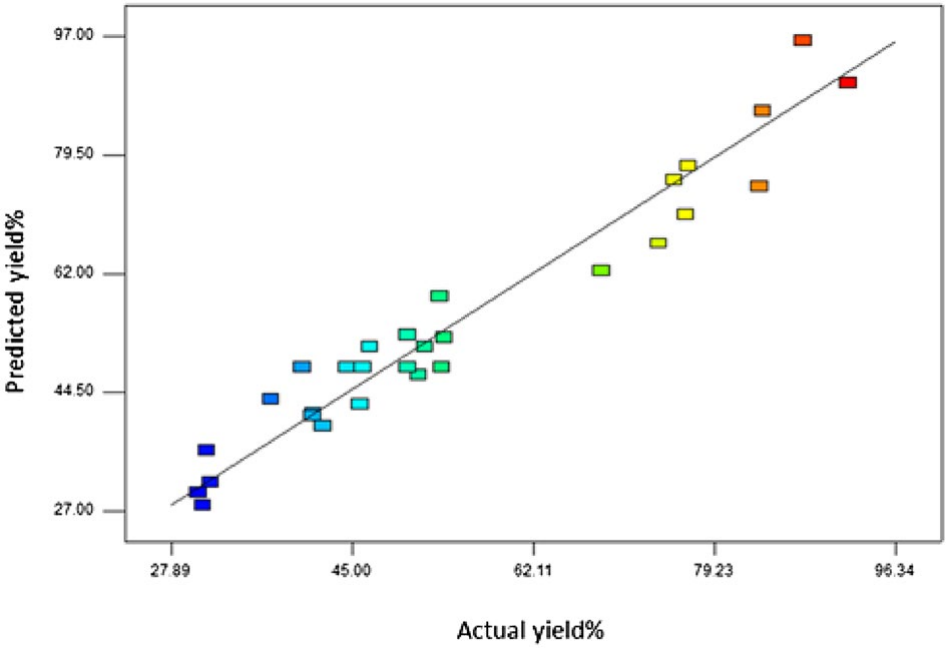
Relationships between the experimental and predicted values.

Another statistical parameter of the model, a coefficient of determination adjusted for the number of parameters in the model relative to the number of points in the design, was 0.9416. This value showed that the model was trustworthy in predicting the response and, at least 94.16% of the variability in the data, could be defined by the second-order polynomial model. The R^2^_pred_ and R^2^_adj_ values were 0.7788 and 0.9109, respectively, that show good fitness of the model because these values are in reasonable agreement with together, as the values should differ by no more than 0.2^72,73^. The adequate precision is a measure of the signal to noise ratio and a quantity greater than 4 is desirable^74^. At this investigation, adequate precision of 20.572 is a good signal to noise ratio and proves the ability of model to navigate the design space (Table 2)^75^. These statistical values along with lack of fit tests display that the quadratic model is adequate to predict the response (the yield of isoamyl acetate (**4**)).The relationship between the experimental values and predicted values are denoted in Fig. 8. It can be seen from this Figure that the experimental values are very close to those predicted using the model equation, showing linear distribution with R^2^ = 0.9416. This value showed that this experimental model is acceptable and reproducible.

**Table 2:** Other statistical parameters of this model.

Std. Dev	5.50	R – Squared	0.9416
Mean	54.54	Adj R-Squared	0.9109
C.V	%10.08	Pred R-Squared	0.7788
PRESS	2174.48	Adeq Precision	20.572

**Figure 9:**
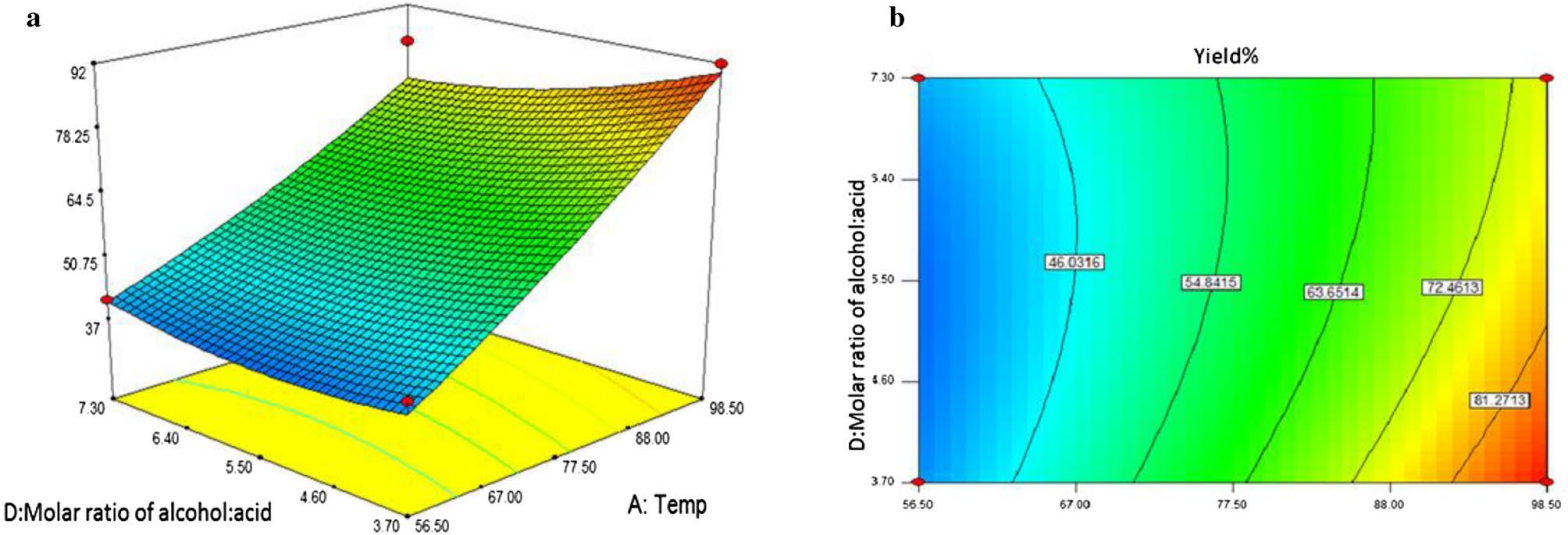
Three-dimensional response surface (**a**) and contour plot for reaction temperature (A) versus molar ratio of acid: alcohol (D) (**b**).

**Figure 10:**
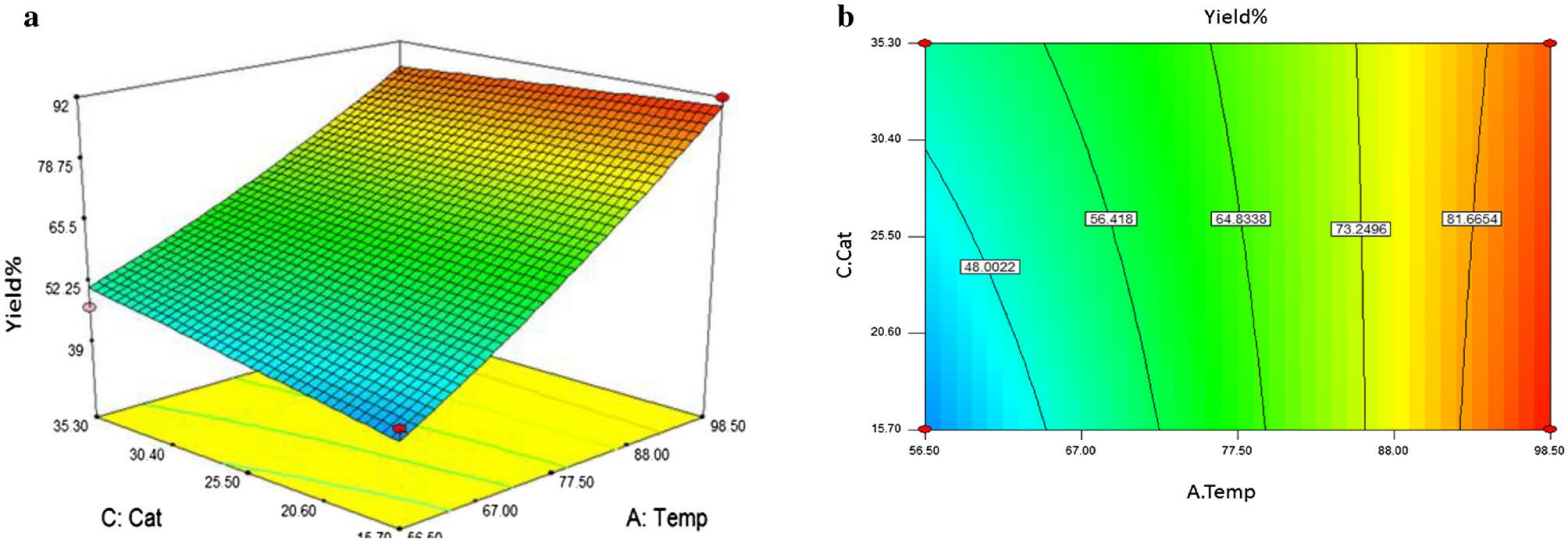
Three-dimensional response surface (**a**) and contour plot for reaction temperature (A) versus catalyst loading (C) (**b**).

The best approach to predict the relationships between responses, variables and interactions is the contour and three-dimensional plots. As shown (Figs. 9, 10), three-dimensional (3D) response surfaces and two-dimensional (2D) contour plots illustrate the effects of different variables on the response. In these plots, two variables are changed, whereas the other variables are kept constant and they describe the type of interactions between two tested variables and the correlations between response and variables levels. 3D response surface and contour plot in Fig. 9 illustrate the influence of interaction between the reaction temperature (A) and molar ratio of acid: alcohol (D) on values of time (B) and catalyst loading (C) were set to 165 min and 25.5 mg, respectively. These results clearly show that at temperature of 56.5 °C, enhancing the molar ratio of reactants had no significant effect on the yield of isoamyl acetate (**4**) whereas at 98 °C lower yields were obtained when higher molar ratio of reactants are used^16,76^. Moreover, Fig. 10 display relationship between reaction temperature (A) and catalyst **3** loading (C). It can also be seen that by increasing the reaction temperature to 98 °C, substantial yields were achieved. However, higher loadings of the catalyst had no significant effect on the yield of reaction.

The main purpose of the response surface methodology (RSM) is detecting the optimal conditions to maximize the percentage of the yield of the favored product (response). For this purpose, four factors were measured in the range of (± 1) to optimize the process while the response (the yield of isoamyl acetate (**4**)) was fixed to a maximum value^77^. According to this method, the optimum values of the factors under solvent-free conditions were 15.7 mg for the catalyst **3** loading, 1:3.7 molar ratio of isoamyl alcohol: acetic acid, 98 °C for the reaction temperature, and 219 min for the reaction time. The yield of the desired product under optimal experimental conditions (91%) is in excellent agreement with the predicted value (89%). Good settings between the experimental and predicted values show the credibility and adequacy of the model to predict the yield of isoamyl acetate (**4**) by esterification of isoamyl alcohol with acetic acid under solvent-free conditions. The crude reaction mixtures were analyzed by gas chromatography (GC) and gas chromatography-mass spectroscopy (GC–MS). The results are shown in the Supplementary Material.

To demonstrate the efficiency of this methodology, Table 3 compares the ability of various heterogeneous catalysts in the esterification reaction of isoamyl alcohol with acetic acid, which represents significant excellence of the ball-milled seashells compared to the most of introduced catalytic systems in terms of catalyst loading, temperature and yield.

**Table 3:** Comparison of the performance of various catalysts versus ball-milled seashells (**3**) in the esterification of isoamyl alcohol (**1**) with acetic acid (**2**).

Entry	Catalyst	Temp (°C)	2:1 mol ratio^a^	Catalyst loading (mg)	Time (min)	Yield %
1	NaX	120	1:1	2000	240	92^78^
2	NaY	120	1:1	2000	240	88^78^
3	β-MnO_2_	124	1:1.8	144	210	92^38^
4	SO_4_^–2^/TiO_2_	130	7:1	3.2^b^	300	94^16^
5	Lipase	30	10:8	12^c^	480	75^20^
6	Lipase	37	2:1	60^d^	480	64^23^
7	Cs(K)_*X*_H_3−*X*_ PW_12_O_40_TPA	180	1;1	500	–	80^79^
8	Ball-Milled seashells	98	3.7:1	15.7	219	91^e^ (this work)

^a^Molar ratio of acid:alcohol.

^b^3.2 wt % with respect to acetic acid.

^c^12% (w/w) enzyme.

^d^60 IU of immobilized lipase.

^e^Average of two runs at optimal conditions.

A plausible mechanism for the reaction of isoamyl alcohol (**1**) and acetic acid (**2**) in the presence of ball-milled seashells (**3**) to afford isoamyl acetate (**4**) is outlined in Fig. 3 (See Supplementary Information). The unique catalytic properties of the ball-milled seashells, as a biodegradable, eco-friendly, and recyclable nano-biocomposite, can be related to synergic effect of the aragonite microcrystalline form with the reinforcing chitin fibers and protein chains compared to the calcite one in commercial CaCO_3_ samples as well as the porosity and hygroscopic properties of the material. According to the proposed mechanism, the oxygen of the carbonyl group of acetic acid (**2**) is activated by the Ca^2+^ species of the seashell (**3**) as well as hydrogen bondings of the OH or even NH groups of chitin fibers and protein chains, respectively, to produce intermediate (**I**)^41,46,57,80–82^. These interactions make the carbonyl group more susceptible to the nucleophilic attack of isoamyl alcohol (**1**) and forming intermediate (**II**). Then, by intermolecular proton transfer and removal of a water molecule, the isoamyl acetate (**4**) is formed.
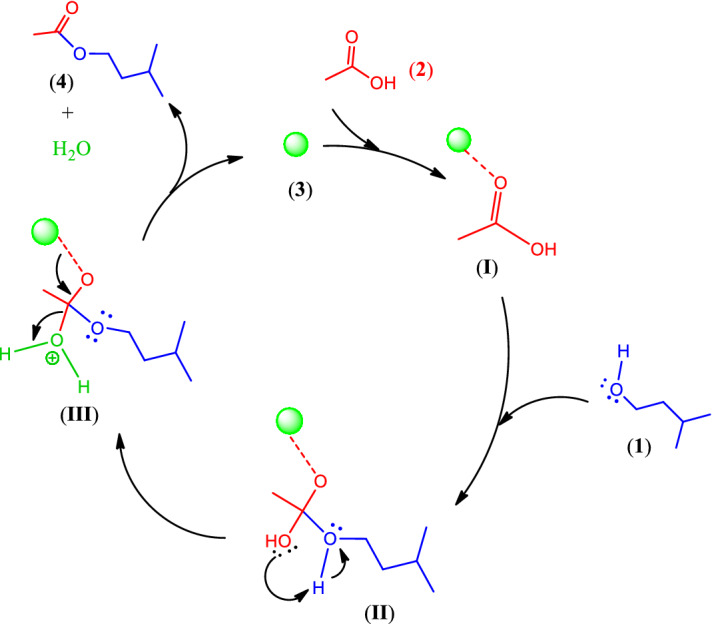


## Experimental section

Isoamyl alcohol and acetic acid were purchased from Merck Chemical Company and used without further purification. The seashells were collected from the southern coast of Caspian Sea, Babolsar, Iran. The ball mill was a Retsch MM 400 swing mill. 10 mL stainless steel ball mill vessels were used for preparation of seashells nano-biocomposite. Two stainless steel balls with 12 mm diameter were used, and the milling frequency was at 25 Hz at the ambient temperature. Yields were obtained using a FID-gas chromatography Shimadzu 2010 instrument equipped with BP5 (30 m, 0.25 mm) column. The oven temperature was maintained at 60 °C, elevated to 200 °C at a rate 10 °C/ min, and was held for 2 min. The injection volume was 1.0 mm^3^. Samples were prepared by adding 0.004 g of *n*-dodecane, as an internal standard, and 5 mL of toluene as solvent. GC–MS chromatograms were recorded on a PerkinElmer Clarus 680 using nitrogen as the carrier gas. FTIR spectrum of the catalyst was obtained using a Shimadzu-8400S spectrometer in the range of 400–4000 cm^−1^ using KBr pellet. SEM images were prepared using a KYKY instrument (model EM-3200). The XRD pattern of the catalyst was obtained using TW 1800 diffractometer with Cu Ka radiation (k = 1.542 Å). Chemical analysis was performed using EDX model Philips XL-30. The Saeshell surface was mounted on the AFM stage and the triboscope recording unit with transducers and leveling device was placed on the top of a NanoScope III E 164 | 164 mm^2^ XY piezo scan base. The tip radii were obtained from direct AFM measurements of the tip apex region in tapping mode. Thermal gravimetric analysis (TGA) was performed by using a Bahr company STA 504 instrument. Statistical analysis and response surface graphs were generated using the Design-Expert 7 software (State Ease Inc., Minneapolis, MN, USA).

### Experimental design and optimization of esterification of acetic acid with isoamyl alcohol by RSM

Optimization of the reaction conditions for the synthesis of isoamyl acetate (**4**) was accomplished using RSM to evaluate the effects of some reaction variables on the yield of the esterification of isoamyl alcohol with acetic acid. In this work, according to the elementary studies and the procured results from the previous studies four variables including reaction temperature (A), reaction time (B), catalyst loading (C) and molar ratio of acetic acid: isoamyl alcohol (D) were chosen as the most effective variables on the yield of the isoamyl acetate (**4**). A 5-level 4-factor RCCCD was employed at this study. Total experimental points of N, there is a combination of factorial (Nf), star (Nα), and central (N0) experiments with 2^F^, 2F and N0 points, respectively. This can be expressed as Eq. 2^16^, in the present study, six experiments were performed in the center of design to allow the estimation of pure error and reproducibility. Hence, the total number of required experiments was 30 (Eq. 1).

2 $${\text{N }} = { 2}^{{\text{F}}} + {\text{2F}} + {\text{ N}}0 = { 16} + {8} + {6} = {3}0$$

The coded and the actual values of the factors are shown in Table 4. The combination of the levels of factors in actual values along with experimental response was presented in Table 5. In this paper, a quadratic-order polynomial model was used to calculate the predicted yield and was fitted in the following equation Eq. 3^58^:

3 $${\mathbf{Y}} = {\text{b}}0 + \sum\limits_{i = 1}^{n} {biXi} + \sum\limits_{i = 1}^{n} {biiXi^{2} } + \sum\limits_{i = 1}^{n} {\sum\limits_{j = i + 1}^{n} {bijXiXj} }$$

where Y is the response (% yield), b0, bi, bii, and bij, are constant, linear, interaction and quadratic coefficients, respectively, and xi, and xj are the coded experimental parameters, which influence on the response. All data were analyzed by analysis of variance (ANOVA) to predict the model and evaluate the significances of the variables and interactions.

**Table 4:** Ranges and levels of the experimental parameters.

Factor	Symbol	− α	− 1	0	+ 1	+ α
Temperature/°C	A	25	56	77	98	130
Time/min	B	30	111	165	219	300
Catalyst loading/mg	C	1.0	15.7	25.7	35.3	50.0
Molar ratio of alcohol: acid	D	1.0	3.7	5.5	7.3	10.0

**Table 5:** RCCCD and experimental data for a 5-level 4-factor response surface methodology.

Run	A^a^	B^a^	C^a^	D^a^	Yield (%)^b^
1	56	219	35.3	3.7	47
2	77	165	25.5	5.5	46
3	98	219	15.7	7.3	84
4	77	165	25.5	5.5	50
5	98	219	15.7	3.7	91
6	77	165	25.5	5.5	50
7	77	165	1.0	3.7	37
8	98	111	15.7	3.7	74
9	98	219	35.3	3.7	84
10	56	219	15.7	7.3	41
11	98	111	35.3	3.7	69
12	98	111	35.3	7.3	51
13	25	165	255	5.5	31
14	130	165	25.5	5.5	88
15	77	165	25.5	5.5	40
16	77	165	50.0	5.5	50
17	56	11	35.3	3.7	41
18	77	165	25.5	5.5	45
19	56	111	15.7	3.7	31
20	77	165	25.5	55	45
21	56	219	35.3	7.3	54
22	56	111	35.3	7.3	46
23	77	165	25.5	1.0	76
24	77	165	25.5	5.5	54
25	77	300	25.5	5.5	77
26	56	111	15.7	7.3	32
27	56	219	15.7	3.7	42
28	98	111	15.7	7.3	52
29	98	219	35.3	7.3	77
30	77	30	25.0	25.5	31

^a^A, B, C, and D parameters are reaction temperature, reaction time, catalyst loading and molar ratio of alcohol: acid, respectively.

^b^Yields were determined by GC analysis.

5.0 g of beach﻿-collected seashells were rinsed thoroughly by distilled water and then heated in refluxing 96% EtOH for 1.0 h to remove any organic impurity. Seashells were removed from EtOH and air dried. Then, the air-dried seashells were washed with water and dried at 70 °C for 1.0 h. The as-treated seashells were put in a ball-mill vessel and milled at 25 Hz frequency for 3.0 min to afford a fine powder. The obtained powder was characterized by common spectroscopic, microscopic and thermal gravimetric analysis.

General procedure for preparation of the ball-milled seashells nano-biocomposite (**3**). The esterification of isoamyl alcohol with acetic acid was performed in a round-bottom flask equipped with a reflux condenser and a magnetic stirrer. Different molar ratios of isoamyl alcohol (**1**) to acetic acid (**2**) and catalyst loadings of ball-milled seashells (**3**) were used at different reaction temperatures and times illustrated in Table 4. During the reaction, the water was eliminated from the reaction mixture by absorbent property of seashells. After completion of the reaction, the obtained mixture was cooled to room temperature. The liquid phase was separated from the catalyst by filtration and analyzed by means of GC and GC–MS instruments. Furthermore, the obtained isoamyl acetate (**4**) was characterized after purification through known solvent extraction procedures by FTIR spectroscopy (See Supplementary Material).

## Conclusion

Pure seashells powder was prepared by ball milling and used, as a multifunctional nano-biocomposite catalyst and natural source of CaCO_3_ in its aragonite microcrystalline form with fixed CO_2_, for the esterification of isoamyl alcohol with acetic acid under environmentally-benign conditions. Also, the reaction conditions for the synthesis of isoamyl acetate, as an industrially important compound, were optimized using response surface methodology with a five-level three-factor rotatable circumscribed central composite design. The obtained results confirmed that RSM is more advantageous than the traditional single parameter optimization in that it saves time, environment and substrates as well as reduces costs substantially. Other advantages of the current protocol include excellent yield, short reaction time, lower catalyst loading and required temperature, the use of an inexpensive, naturally occurring and easily prepared nano-biocomposite material having appropriate thermal stability and working under solvent-free conditions as well as no use of corrosive Bronsted acids, toxic azeotropic solvents or water adsorbents, and simplicity of the procedures for both nano-biocomposite preparation and isoamyl acetate synthesis. To the best of our knowledge, this is the first report on the catalytic activity of the pure ball-milled seashells for organic transformations. Hence, other organic reactions including named multi-component reactions promoted by ball-milled seashells have been investigated in our research group and the results will be published in due course.

## Supplementary Information

Supplementary Information.

## Data Availability

All data generated or analyzed during this study are included in this published article [and its supplementary information files].
