# Supplementary material for: Optimization of green and environmentally-benign synthesis of isoamyl acetate in the presence of ball-milled seashells by response surface methodology
Source: Sci Rep. 2023 Feb 16;13:2803. doi: 10.1038/s41598-023-29568-y (PMC9935880; doi:10.1038/s41598-023-29568-y)
Supplement: Supplementary file 1 — Supplementary Information. [file 41598_2023_29568_MOESM1_ESM.docx]

**Optimization of green and environmentally-benign synthesis of isoamyl acetate in the presence of ball-milled seashells by response surface methodology**

*Amir Hossein Fattahi^1^, Mohammad G. Dekamin^1*^ and James H. Clark^2^*

*^1^Pharmaceutical and Heterocyclic Compounds Research Laboratory, Department of Chemistry, Iran University of Science and Technology, Tehran, 16846-13114, Iran*

*^2^* *Green Chemistry Centre of Excellence, Department of Chemistry, University of York, York, YO10 5DD, UK*

*.*

**E-mail: mdekamin@iust.ac.ir*

| **Page** | **Contents** |
| --- | --- |
| S1 | Title page |
| S2 | Experimental section (General) |
| S2 | General procedure for preparation of the ball-milled seashells nano-biocomposite (**3**) |
| S2 | General procedure for the esterification of isoamyl alcohol (**1**) with acetic acid (**2**) catalyzed by ball-milled seashells nano-biocomposite (**3**) |
| S3 | **Fig. 1**. Chromatogram of the crude reaction mixture for the synthesis of isoamyl acetate (**4**) under optimized conditions in GC-MS analysis. |
| S4 | **Fig. 2.** Mass spectrum of the reaction mixture for synthesis isoamyl acetate (**4**) catalyzed by ball-milled seashells nano-biocomposite (**3**). |
| S5 | **Fig. 3.** Proposed mechanism for the synthesis of isoamyl acetate (**4**) from isoamyl alcohol (**1**) and acetic acid (**2**) in the presence of ball-milled seashells (**3**). |

Experimental section

General

### Isoamyl alcohol and acetic acid were purchased from Merck Chemical Company and used without further purification. The seashells were collected from the southern coast of Caspian Sea, Babolsar, Iran. The ball mill was a Retsch MM 400 swing mill. 10 mL stainless steel ball mill vessels were used for preparation of seashells nano-biocomposite. Two stainless steel balls with 12 mm diameter were used, and the milling frequency was at 25 Hz at the ambient temperature. Yields were obtained using a FID-gas chromatography Shimadzu 2010 instrument equipped with BP5 (30 m, 0.25 mm) column. The oven temperature was maintained at 60 °C, elevated to 200 °C at a rate 10 °C/ min, and was held for 2 min. The injection volume was 1.0 mm^3^. Samples were prepared by adding 0.004 g of n-dodecane, as an internal standard, and 5 mL of toluene as solvent. GC-MS chromatograms were recorded on a PerkinElmer Clarus 680 using nitrogen as the carrier gas. FTIR spectrum of the catalyst was obtained using a Shimadzu-8400S spectrometer in the range of 400–4000 cm^-1^ using KBr pellet. SEM images were prepared using a KYKY instrument (model EM-3200). The XRD pattern of the catalyst was obtained using TW 1800 diffractometer with Cu Ka radiation (k = 1.542 Å). Chemical analysis was performed using EDX model Philips XL-30. The tip radii were obtained from direct AFM measurements of the tip apex region in tapping mode. The Saeshell surface was mounted on the AFM stage and the triboscope recording unit with transducers and leveling device was placed on the top of a NanoScope III E 164 | 164 mm2 XY Piezo scan base. Thermal gravimetric analysis (TGA) was performed by using a Bahr company STA 504 instrument. Statistical analysis and response surface graphs were generated using the Design-Expert 7 software (State Ease Inc., Minneapolis, MN, USA).

### **General procedure for preparation of the ball-milled seashells nano-biocomposite (3)**

### 5.0 g of beach-collected seashells were rinsed thoroughly by distilled water and then heated in refluxing 96% EtOH for 1.0 h to remove any organic impurity. Seashells were removed from EtOH and air dried. Then, the air-dried seashells were washed with water and dried at 70 °C for 1.0 h. The as-treated seashells were put in a ball-mill vessel and milled at 25 Hz frequency for 3.0 min to afford a fine powder. The obtained powder was characterized by common spectroscopic, microscopic and thermal gravimetric analysis.

### **General procedure for the esterification of isoamyl alcohol (1) with acetic acid (2) catalyzed by ball-milled seashells nano-biocomposite (3)**

The esterification of isoamyl alcohol with acetic acid was performed in a round-bottom flask equipped with a reflux condenser and a magnetic stirrer. Different molar ratios of isoamyl alcohol (**1**) to acetic acid (**2**) and catalyst loadings of ball-milled seashells were used at different reaction temperatures and times illustrated in **Table 4**. During the reaction, the water was eliminated from the reaction mixture by absorbent property of seashells. After completion of the reaction, the obtained mixture was cooled to room temperature. The liquid phase was separated from the catalyst by filtration and analyzed by means of GC and GC–MS instruments. Furthermore, the obtained isoamyl acetate (**4**) was characterized after purification through known solvent extraction procedures by FTIR spectroscopy (See Supplementary Material).


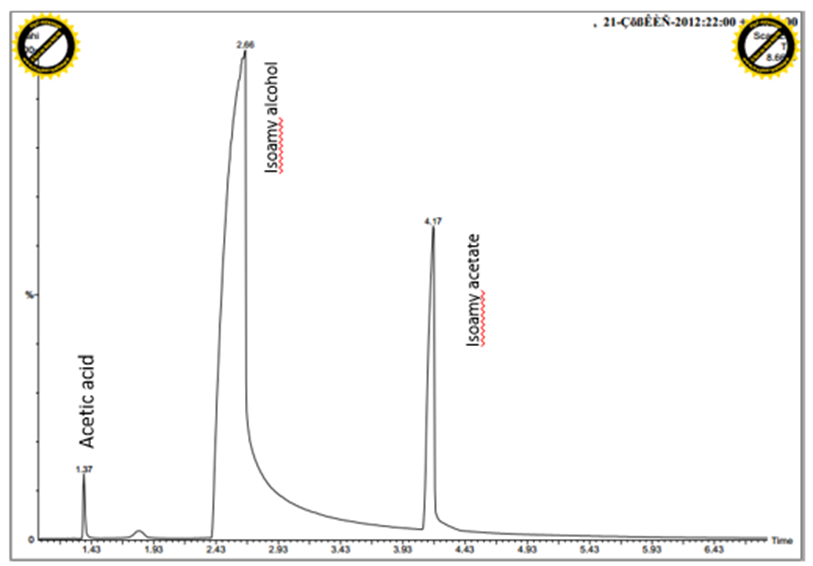


**Fig. 1**. Chromatogram of the crude reaction mixture for the synthesis of isoamyl acetate (**4**) under optimized conditions in GC-MS analysis.

| 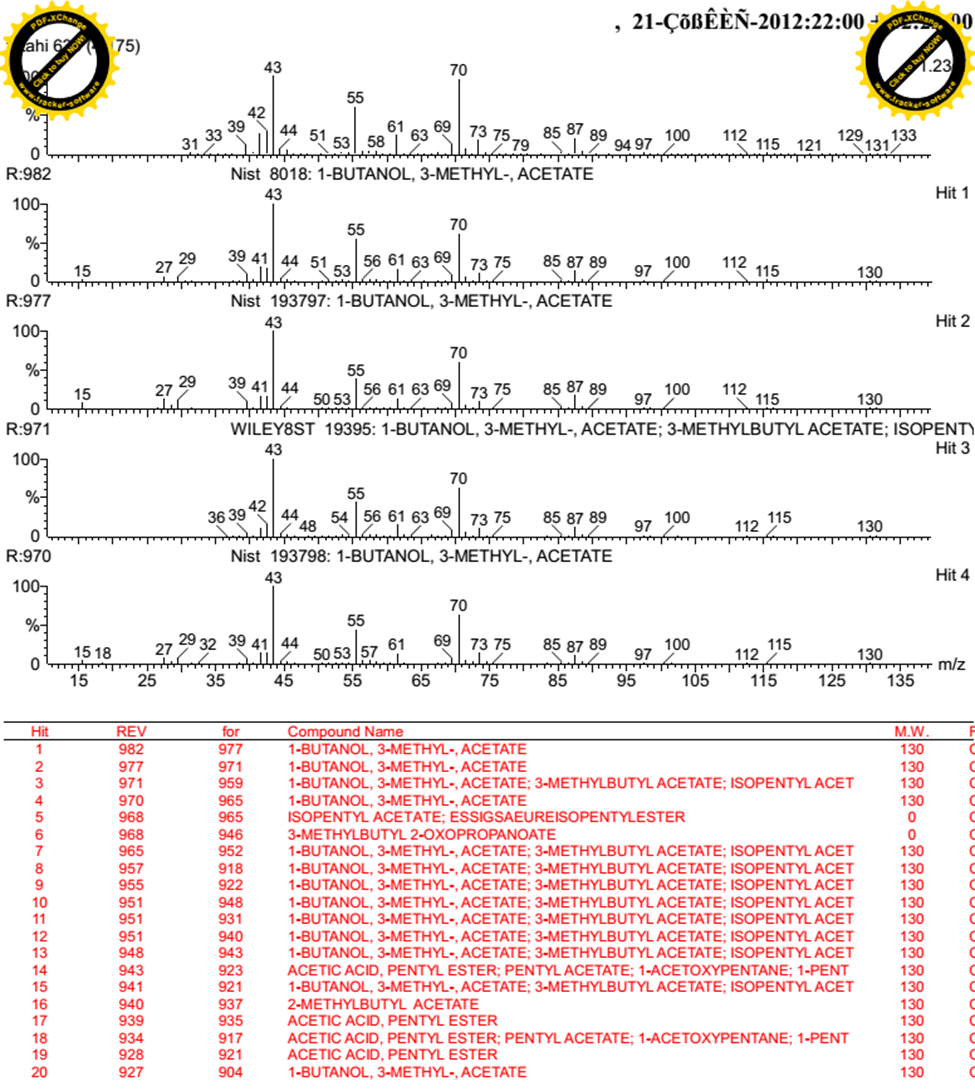  **Fig. 2.** Mass spectrum of the reaction mixture for synthesis isoamyl acetate (**4**) catalyzed by ball-milled seashells nano-biocomposite (**1**). |
| --- |

**Fig. 3.** Proposed mechanism for the synthesis of isoamyl acetate (**4**) from isoamyl alcohol (**1**) and acetic acid (**2**) in the presence of ball-milled seashells (**3**).
